# A Systematic Review of Teachers’ Causal Attributions: Prevalence, Correlates, and Consequences

**DOI:** 10.3389/fpsyg.2018.02305

**Published:** 2018-12-17

**Authors:** Hui Wang, Nathan C. Hall

**Affiliations:** ^1^Department of Special Education and Counselling, The Education University of Hong Kong, Tai Po, Hong Kong; ^2^Department of Educational and Counselling Psychology, McGill University, Montreal, QC, Canada

**Keywords:** teachers, causal attributions, interpersonal, intrapersonal, occupational stress, review

## Abstract

The current review provides an overview of published research on teachers’ causal attributions since 1970s in the context of theoretical assumptions outlined in Weiner’s (2010) attribution theory. Results across 79 studies are first examined with respect to the prevalence of teachers’ interpersonal causal attributions for student performance and misbehavior, as well as intrapersonal attributions for occupational stress. Second, findings showing significant relations between teachers’ attributions and their emotions and cognitions, as well as student outcomes, are discussed. Third, an overview of results showing the prevalence and implications of teachers’ causal attributions to be moderated by critical background variables is also provided. Finally, observed themes across study findings are highlighted with respect to the fundamental attribution error and the utility of Weiner’s attribution theory for understanding how teachers’ explanations for classroom stressors impact their instruction, well-being, and student development.

## Introduction

Classroom instruction and learning are complex processes characterized by intensive cognition as to the causal nature of student and teacher outcomes. When a student performs poorly on an academic task, for example, he or she might ascribe the failure to lack of effort, bad luck, or teaching quality. Likewise, teachers also experience successes and failures with respect to their instructional goals concerning student performance and classroom behaviors, with studies showing teachers who fail to achieve their personal teaching goals to report a more maladaptive emotional profile (Sutton, 2007; Frenzel et al., 2009, 2015; Frenzel, 2014) as well as poorer levels of occupational stress, burnout, satisfaction, and commitment (Chang, 2009). Recent findings further suggest that teachers’ motivational beliefs may help mitigate the impact of perceived instructional failures on their emotional well-being such as intrinsic motivation (e.g., Roth et al., 2007; Roness, 2011), achievement goal orientations (e.g., Butler, 2012; Wang et al., 2017), and teaching self-efficacy (Klassen et al., 2011).

Over the past 50 years, motivation research has also consistently examined the effects of individuals’ causal explanations or attributions for failure experiences on their emotions, persistence, and achievement in educational settings (e.g., Heider, 1958; Atkinson, 1964). According to attribution theory, individuals are particularly motivated to seek specific explanations for negative educational outcomes, with these causal attributions, in turn, having important consequences for academic development (Weiner, 1985, 2010). Whereas most research applying attribution theory to the classroom has focussed on students’ causal attributions for academic setbacks (for reviews, see Weiner, 1976; Wilson and Linville, 1982; Ames and Ames, 1984; Graham, 1984b, 1991; Forsterling, 1985; Graham and Williams, 2009), the nature and effects of teachers’ causal attributions for their classroom experiences has also received consistent empirical attention (Cooper and Lowe; 1977, study 2; Tollefson and Chen, 1988; Butler, 1994; Matteucci et al., 2008b; Wang et al., 2015). However, there to date exists no comprehensive review article in education literature of empirical research on the prevalence, correlates, and consequences of causal attributions for classroom challenges made by teachers. To address this unfortunate research gap, the present article provides a systematic review of published research on the types of attributions made by practicing teachers’ when faced with student problems and teaching obstacles as well as their impact on instructional behaviors, student outcomes, and teachers’ own psychological well-being.

## Conceptual Framework

### Attribution Theory

According to Weiner’s (1985, 2000, 2010) attribution theory, a causal attribution is defined as an individual’s perception as to the cause of a success or failure event, with the attribution selected able to be classified according to underlying causal dimensions that correspond to specific effects on subsequent emotions, decision-making, and performance. The first of these causal dimensions, *locus of causality*, refers to the attribution being either internal or external to the individual in origin. For example, attributions having an internal locus of causality would prototypically implicate one’s own ability or effort, whereas attributions to external factors might instead involve luck or environmental circumstances. In contrast, the second causal dimension, *stability*, reflected the extent to which one attributed an event to causes that were temporally persistent, such as low ability, or causes that fluctuated over time, such as luck. In the event of failure, although one would expect it to reoccur if an attribution was made to a stable antecedent, attributions to unstable causes should be more motivating in allowing for the possibility of future success.

Although Weiner’s initial model included only two causal dimensions, a third dimension referred to as *controllability* – the extent to the perceived cause of an outcome is regarded as personally changeable – was later added to distinguish this framework from the “locus of control” construct proposed by Rotter (1966; Weiner, 1979). In contrast to Rotter’s model in which the concepts of internality and controllability were conflated (e.g., internal locus: attributions to effort and ability), Weiner differentiated between them given the divergent motivational consequences of internal attributions depending on their perceived controllability. For example, whereas a failure attribution to lack of effort implies the potential for improvement, attributing failure to an intractable lack of ability does not.

In addition to differentiating three dimensions underlying causal attributions, attribution theory further proposes specific cognitive and emotional consequences of each dimension that occur after causal attributions are selected, namely expectations of future performance and perceived responsibility for the events. Whereas attributions with an internal *locus of causality* should lead to perceptions of self-esteem and feelings of pride, expectations for future success and the emotions of hopefulness/hopelessness should be more significantly impacted by *stability* dimension of the attribution selected (Weiner, 1985, 2000, 2010). Likewise, the *controllability* dimension is proposed to most significantly correspond with perceptions of personal responsibility and the emotions of guilt and shame. This theory further proposes the occurrence of mixed emotions, for example, with failure attributions to lack of effort contributing to less pride (internal) yet higher levels of motivating emotions such as hope (unstable) and guilt (controllable), emotions that, in turn, are assumed to directly contribute to achievement-striving behaviors and performance.

### Interpersonal and Intrapersonal Applications of Weiner’s Attribution Theory

Weiner’s (2001) attribution theory also differentiates between intrapersonal and interpersonal attributions for achievement outcomes. The intrapersonal perspective, as described above, refers to the attributions individuals make for their own performance, focusing on how expectations for personal success and responsibility can lead to self-directed feelings of pride, guilt, hope, or shame and, in turn, self-relevant educational outcomes (e.g., persistence). On the other hand, the interpersonal approach to Weiner’s theory concerns the attributions made for outcomes experienced by others and focuses primarily on how perceptions of another’s responsibility for an outcome contributes to other-directed emotions (e.g., sympathy, anger) and behaviors (e.g., punishment, assistance; Weiner, 2001, 2003). For example, although teachers may attribute their own instructional failures or occupational stress to specific factors (e.g., insufficient resources, lesson preparation), their attributions for students’ misbehaviors or poor performance may differ (e.g., insufficient student effort, parental support).

With respect to teacher behavior resulting from attributions for student failure to factors implicating student responsibility, Weiner (2003, 2010) further proposed two main goals underlying teacher reprimands: *utilitarian goals* or *retributive goals*. In contrast to teachers with utilitarian goals who use punishment to encourage future behavioral change, teachers with retributive goals focus instead on students’ past transgressions and administering negative deserved consequences out of fairness (Weiner, 2000). Moreover, early scenario research by Weiner and Kukla (1970) suggested that although the degree of reward vs. punishment administered by teachers depended largely on student performance, the perceived combination of prototypic attributions (student effort vs. ability) could also impact teaching behavior. In this study, poor student performance attributed to high ability combined with lack of effort was most strongly punished, whereas poor-performing students perceived as investing effort despite low ability were not punished as severely. Follow-up research by Reyna and Weiner (2001) further indicated that retributive punishment was more likely when students were perceived as responsible for their failure due to lack of effort, whereas more utilitarian punishment was reported when students were believed to lack ability and not be responsible for their performance (Weiner, 2003).

### The Present Review

As outlined above, Weiner’s (1985, 2000, 2001, 2010) attribution theory thus represents a comprehensive theoretical framework for understanding how individuals in educational settings perceive the causes underlying the performance of themselves and others, as well as the effects of these causal attributions on subsequent cognitions, emotions, and behaviors in the classroom. In incorporating both interpersonal and intrapersonal perspectives, this theory is also particularly useful for understanding how teachers perceive student challenges and occupational stressors, and how their attributions for these experiences impact student–teacher interactions, teaching behaviors, and teachers’ emotional well-being. However, despite multiple publications providing in-depth reviews of research on causal attributions in students since the 1970s (e.g., Weiner, 1972, 1976; Graham, 1984b, 1991; Graham and Williams, 2009), there to date exists no published comprehensive reviews of research on causal attributions in teachers. The current review attempts to address the gap in motivation research literature by reviewing existing published research specifically exploring the types of causal attributions made by practicing K-12 teachers for both student difficulties and personal failures. More specifically, the current review aimed to answer: (1) how prevalent are certain attributional styles in teachers and (2) what are the relevant antecedents, correlates, and consequences of certain attributional styles on teachers and their students. Implications of the findings presented concerning future directions in teacher motivation and professional development practices will also be discussed. In the sections that follow, we first present our literature search and screening protocols, followed by subsequent reviews attributions of an interpersonal nature (concerning students’ academic performance vs. misbehaviors) and of an intrapersonal nature (teachers’ own occupational stress). Each type of causal attribution style is further described in each section with respect to its prevalence, relationships with teacher and/or student outcomes, and the extent of observed disagreement between teachers and others (e.g., parents, students) concerning specific attributional styles.

## Methods

**FIGURE 1:**
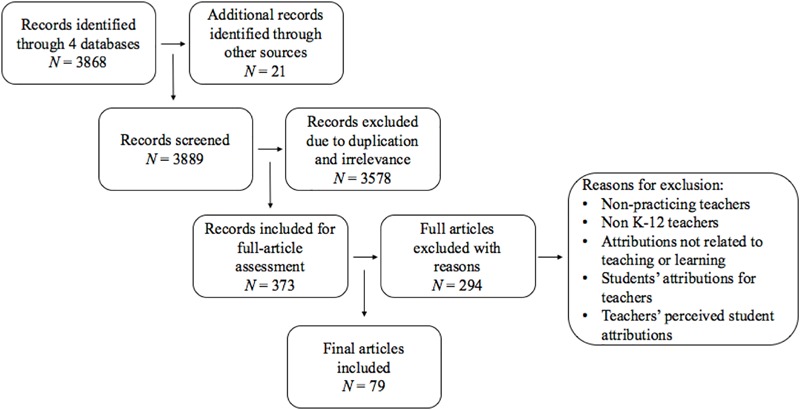
Literature searches flow diagram.

Four databases were included in the literature search including ERIC, PsycINFO, Web of Science, and Scopus. Concerning article search terms, the word “teacher” or “instructor” or “educator” have been included with the words “attribution” or “attributing” or “causal explanation” or “causal ascription” also being included in the keywords or abstract. Database searches included journal articles (e.g., empirical articles, reviews, open-access), book chapters, doctoral dissertations, or conference proceedings written in the English language (see Table 1 and Figure 1 for details on search terms and results). Snowball searches were also conducted by reviewing references of primary articles found through database searches. From an initial total of more than 3,889 search results, 373 unique and relevant articles were subsequently screened as per four exclusion criteria specific to the aim of the current review to investigate causal attributions in practicing K-12 educators concerning their teaching practice.

**Table 1:** Key search term.

Database	Search strategy	Results
Eric ProQuest	Teacher^∗^ AND attribution^∗^	341
	Instructor^∗^ AND attribution^∗^	27
	Educator^∗^ AND attribution^∗^	35
	Teacher^∗^ AND attributing	43
	Instructor^∗^ AND attributing	4
	Educator^∗^ AND attributing	12
	Teacher^∗^ AND causal explanation^∗^	35
	Instructor^∗^ AND causal explanation^∗^	2
	Educator^∗^ AND causal explanation^∗^	6
	Teacher^∗^ AND causal ascription^∗^	2
	Instructor^∗^ AND causal ascription^∗^	0
	Educator^∗^ AND causal ascription^∗^	0
	Total	507
PsycINFO	Teacher^∗^ AND attribution^∗^	677
	Instructor^∗^ AND attribution^∗^	47
	Educator^∗^ AND attribution^∗^	71
	Teacher^∗^ AND attributing	35
	Instructor^∗^ AND attributing	1
	Educator^∗^ AND attributing	10
	Teacher^∗^ AND causal explanation^∗^	12
	Instructor^∗^ AND causal explanation^∗^	0
	Educator^∗^ AND causal explanation^∗^	3
	Teacher^∗^ AND causal ascription^∗^	3
	Instructor^∗^ AND causal ascription^∗^	0
	Educator^∗^ AND causal ascription^∗^	0
	Total	859
Web of Science	Teacher^∗^ AND attribution^∗^	738
	Instructor^∗^ AND attribution^∗^	59
	Educator^∗^ AND attribution^∗^	91
	Teacher^∗^ AND attributing	47
	Instructor^∗^ AND attributing	5
	Educator^∗^ AND attributing	11
	Teacher^∗^ AND causal explanation^∗^	87
	Instructor^∗^ AND causal explanation^∗^	10
	Educator^∗^ AND causal explanation^∗^	12
	Teacher^∗^ AND causal ascription^∗^	4
	Instructor^∗^ AND causal ascription^∗^	0
	Educator^∗^ AND causal ascription^∗^	0
	Total	1,064
Scopus	Teacher^∗^ AND attribution^∗^	956
	Instructor^∗^ AND attribution^∗^	108
	Educator^∗^ AND attribution^∗^	152
	Teacher^∗^ AND attributing	77
	Instructor^∗^ AND attributing	4
	Educator^∗^ AND attributing	15
	Teacher^∗^ AND causal explanation^∗^	92
	Instructor^∗^ AND causal explanation^∗^	11
	Educator^∗^ AND causal explanation^∗^	15
	Teacher^∗^ AND causal ascription^∗^	8
	Instructor^∗^ AND causal ascription^∗^	0
	Educator^∗^ AND causal ascription^∗^	0
	Total	1,438

*^∗^is a wildcard symbol that broadens the search by including all terms having these specific root letters.*

First, studies with non-practicing teachers were excluded, including studies with pre-service teachers (e.g., Goyette et al., 2000; Shields and Edens, 2009; Carter et al., 2014), teaching assistants (e.g., Morgan and Hastings, 1998; Meel, 2011), and convenience samples (e.g., psychology undergraduates; Weiner and Kukla, 1970; Reyna and Weiner, 2001, study 1). Second, as the review focuses on attributions made by K-12 teachers, studies with teachers in other educational contexts such as pre-school teachers (e.g., Dobbs and Arnold, 2009), vocational or language teachers outside K-12 settings (e.g., Dubois, 2000, study 3; Ghanizadeh and Ghonsooly, 2014, 2015; Jonasson, 2014), and post-secondary instructors were excluded (e.g., Ames, 1975; Zhou, 2006).

Third, articles examining attributions made by teachers that were not related to teaching or learning were excluded, such as attributions related to students’ physical attributes (e.g., Felson, 1980; Roth and Eisenberg, 1983), students’ unhealthy lifestyles (e.g., Power et al., 2010), chronic pain (Logan et al., 2007), poverty (e.g., Robinson, 2011), teacher selection and screening (e.g., Pounder, 1987), or principals’ behaviors (e.g., Leithwood and Jantzi, 1997). Finally, studies in which attributions made by students concerning their teachers were excluded (e.g., Wyatt and Haskett, 2001; Chedzoy and Burden, 2009), as were articles exploring students’ perceived teacher attributions (e.g., Hau and Salili, 1996; Prihadi et al., 2012) or vice versa (e.g., Bar-Tal et al., 1981), to more clearly demarcate attributions made by teachers from those made by students. With the exclusion criteria applied, the final review total included 79 empirical papers in which practicing teachers’ causal attributions for instructional issues were explicitly addressed with each article selected aligning with one of three emergent themes: teacher attributions for student performance, student behavioral/emotional problems, and teachers’ occupational stress (see Table 2 for details on study designs and paradigms for all these studies and see Table 3 for an overview of study outcomes, domains, and dimensions).

**Table 2:** Study designs and paradigms for all 79 studies reviewed.

Reference	Sample size	Grade level	Country/region	Type of study	Cross-sectional	Measurement for attribution	Dimensions of attributions
1. Algozzine and Stoller, 1981	46 teachers	Special education	United States	Survey	Yes	Self-developed questionnaire	Academic performance
2. Andreou and Rapti, 2010	249 teachers	Elementary	Greece	Survey	Yes	Mavropoulou and Padeliadu, 2002	Student misbehaviors
3. Arbeau and Coplan, 2007	202 teachers	Kindergarten	Canada	Survey	Yes	Hastings and Coplan, 1999; Coplan et al., 2002	Student misbehaviors
4. Arcia et al., 2000	21 teachers	Elementary	United States	Interview	No (two interviews)	Semi-structured interviews	Student misbehaviors
5. Bar-Tal and Guttmann, 1981	8 teachers, 69 pupils, and 69 parents	Elementary	Israel	Survey	Yes	Questionnaire adapted from Bar-Tal and Darom (1979)	Academic performance
6. Beckman, 1976	9 teachers and 49 parents	Elementary	United States	Survey	Yes	Self-developed questionnaire	Academic performance
7. Bertrand and Marsh, 2015	19 teachers	Elementary and secondary	United States	Interview, survey	No (three interviews)	Semi-structured interviews and self-developed questionnaire	Academic performance
8. Bibou-Nakou et al., 1999	200 teachers	Elementary	Greece	Survey	Yes	Self-developed questionnaire	Student misbehaviors
9. Bibou-Nakou et al., 2000	200 teachers	Elementary	Greece	Survey	Yes	Self-developed questionnaire	Student misbehaviors
10. Brady and Woolfson, 2008	118 teachers	Elementary	United Kingdom	Survey	Yes	Woolfson et al., 2007	Academic performance
11. Burger et al., 1982	17 teachers	Elementary	United States	Survey	No (three time points)	Cooper and Burger, 1980	Academic performance
12. Butler, 1994	60 teachers (Study 1)	Elementary	Israel	Survey	Yes	Self-developed questionnaire	Academic performance
13. Christenson et al., 1983	105 teachers	Elementary	United States	Survey	No (each referral occurrence)	Self-developed questionnaire	Student misbehaviors
14. Clark, 1997	97 teachers	Elementary	United States	Survey	Yes	Self-developed questionnaire	Academic performance
15. Conway, 1989	5 teachers	Not specified	United Kingdom	Content analysis	Yes	Students’ end-of-year reports	Academic performance
16. Cooper and Burger, 1980	39 teachers (Study 1)	Elementary, secondary, and special education	United States	Survey	Yes	Self-developed questionnaire	Academic performance
17. Cooper and Lowe, 1977	8 teachers (Study 2)	Elementary	United States	Survey	Yes	Self-developed questionnaire	Academic performance
18. Cothran et al., 2009	23 teachers and 182 student	Secondary	United States	Interview	Yes	Patton, 2002	Student misbehaviors
19. Ding et al., 2010	244 teachers	Elementary and secondary	China	Survey	Yes	Self-developed questionnaire	Student misbehaviors
20. Emmerich et al., 2006	1,792 teachers	Elementary and secondary	United States	Survey	Yes	Emmerich et al., 2004	Academic performance
21. Espinoza et al., 2014	64 teachers	Secondary	United States	Experiment	No (four phases)	Self-designed questionnaire	Academic performance
22. Fennema et al., 1990	38 teachers	Elementary	United States	Interview and survey	Yes	Structured interview and adapted questionnaire from Broverman et al. (1970)	Academic performance
23. Georgiou, 2008	154 teachers and 159 student teachers	Elementary	Cyprus	Survey	Yes	Georgiou et al., 2002b	Academic performance
24. Georgiou et al., 2002a	277 teachers	Elementary	Cyprus	Survey	Yes	Scale adapted from O’Sullivan and Howe (1996)	Academic performance
25. Gibbs and Gardiner, 2008	455 teachers	Elementary and secondary	England and Ireland	Survey	Yes	Scale adapted from Miller et al. (2000, 2002)	Student misbehaviors
26. Gosling, 1994	160 teachers	Secondary	France	Experiment	Yes	Self-developed questionnaires	Academic performance
27. Graham, 1984a	2 teacher experimenters and 176 students	Elementary	United States	Experiment	Yes	Self-developed questions	Academic performance
28. Guskey, 1982	184 teachers	Elementary and secondary	United States	Survey	Yes	Self-developed questionnaire	Academic performance
29. Guttmann, 1982	28 teachers, 107 mothers, and 220 students	Elementary	Israel	Survey	Yes	Self-developed questionnaire	Student misbehaviors
30. Hall et al., 1989	214 teachers	Elementary and secondary	United States	Survey	Yes	Self-developed questionnaire	Academic performance
31. Ho, 2004	473 teachers	Secondary	Australia and Hong Kong	Survey	Yes	Self-developed questionnaire	Student misbehaviors
32. Hughes et al., 1993	55 teachers	Elementary	United States	Survey and Interview	Yes	Medway, 1979	Student misbehaviors
33. Hui, 2001	214 teachers and 2,045 students	Secondary	Hong Kong	Survey	Yes	Hui, 2001, 1997 Unpublished	Student misbehaviors
34. Jackson, 2002	64 teachers, 3 school administrators, and 6 professionals	Elementary	United States	Content analysis	Yes	School referral forms	Student misbehaviors
35. Jager and Denessen, 2015	64 teachers	Secondary	Netherlands	Survey	Yes	Self-developed questionnaire	Academic performance
36. Juvonen, 1988	4 teachers and 84 students	Elementary	Finland	Survey	Yes	Self-developed questionnaire	Academic performance
37. Kauppi and Porhola, 2012	215 teachers	Elementary and secondary	Finland	Survey	Yes	Self-developed questionnaire	Student misbehaviors
38. Klassen and Lynch, 2007	7 teachers and 28 students	Secondary, special education	Canada	Interview	Yes	Semi-structured questionnaire	Academic performance
39. Kleftaras and Didaskalou, 2006	35 teachers and 323 students	Elementary	Greece	Survey	Yes	Self-developed questionnaire	Student misbehaviors
40. Kulinna, 2007	199 teachers	Elementary and secondary	United States	Survey	Yes	Self-developed questionnaire	Student misbehaviors
41. Kurtz et al., 1990	102 teachers	Elementary	Germany and United States	Survey	Yes	Questionnaire adapted from Carr et al. (1989)	Academic performance
42. Matteucci, 2007	Study 1: 122 teachers; Study 2: 202 teachers	Secondary	Italy	Survey	Yes	Self-developed questionnaire extended upon and adapted from Reyna and Weiner (2001)	Academic performance
43. Matteucci and Gosling, 2004	Study 1: 115 teachers; Study 2: 118 teachers	Secondary	Study 1: Italy; Study2: Italy and France	Survey	Yes	Self-developed questionnaire	Academic performance
44. Matteucci et al., 2008b	126 teachers	Elementary	Italy	Survey	Yes	Questionnaire adapted from Dubois and Le Poultier (1991)	Academic performance
45. Mavropoulou and Padeliadu, 2002	305 teachers	Elementary	Greece	Survey	Yes	Questionnaire adapted from Soodak and Podell (1994)	Student misbehaviors
46. McAuliffe et al., 2009	12 teachers and 127 students	Elementary	United States	Classroom observation, survey	Yes	Johnston et al., 2000	Student misbehaviors
47. McCormick, 1997	487 teachers	Elementary and secondary	Australia	Survey	Yes	Self-developed questionnaire	Occupational stress
48. McCormick and Shi, 1999	687 teachers	Elementary and secondary	Australia and China	Survey	Yes	Self-developed questionnaire	Occupational stress
49. McCormick and Solman, 1992a	387 teachers	Elementary and secondary	Australia	Survey	Yes	Self-developed questionnaire	Occupational stress
50. McCormick and Solman, 1992b	111 teachers	Elementary and secondary	Australia	Survey	Yes	Self-developed questionnaire	Occupational stress
51. Medway, 1979	Study 1: 30 teachers; Study 2: 24 teachers	Study 1: Elementary and secondary; Study 2: Elementary	United States	Study 1: Interview; Study 2: Survey	Yes	Self-developed questionnaire	Student learning and behaviors
52. Miller, 1995	24 teachers	Elementary	United Kingdom	Interview	Yes	Structured interview	Student misbehaviors
53. Natale et al., 2009	16 teachers and 69 students	Kindergarten	Finland	Survey	No (two assessments)	Self-developed questionnaire	Academic performance
54. Pirrone, 2012	370 teachers	Elementary	Italy	Survey	Yes	Questionnaire adapted from Rousvoal (1987)	Academic performance
55. Poulou and Norwich, 2000	391 teachers	Elementary	Greece	Survey	Yes	Self-developed questionnaire	Student misbehaviors
56. Prawat et al., 1983	58 teachers	Elementary	United States	Survey	Yes	Self-developed questionnaire	Academic performance
57. Rejeski and McCook, 1980	40 teachers	Elementary	United States	Survey	Yes	Questionnaire adapted from Lowe et al. (1978)	Academic performance
58. Reyna and Weiner, 2001	40 teachers (Study 2)	Secondary	United States	Survey	Yes	Self-developed questionnaire	Academic performance
59. Riley and Ungerleider, 2012	21 teachers	Secondary	Canada	Interview	Yes	Self-developed questionnaire	Academic performance
60. Rodriquez and Tollefson, 1987	155 teachers	Elementary	Costa Rica	Survey	Yes	Self-developed questionnaire	Academic performance
61. Rolison and Medway, 1985	180 teachers	Elementary	United States	Experiment	Yes	Self-developed questionnaire	Academic performance
62. Ross et al., 1974	32 teachers and 32 students	Elementary and secondary	United States	Experiment	Yes	Self-developed questionnaire	Academic performance
63. Savina et al., 2014	80 teachers, 30 psychologists, and 99 parents	Elementary	Russia	Survey	Yes	Self-developed questionnaire	Student misbehaviors
64. Soodak and Podell, 1994	110 teachers	Elementary	United States	Survey	Yes	Self-developed questionnaire	Student learning and misbehaviors
65. Tõeväli and Kikas, 2016	53 teachers and 760 students	Elementary	Estonia	Survey	No (two assessments)	Questionnaire adapted from Natale et al. (2009) and Upadyaya et al. (2012)	Academic performance
66. Tollefson and Chen, 1988	161 teachers	Elementary and secondary	United States	Survey	Yes	Self-developed questionnaire	Academic performance
67. Tollefson et al., 1991	97 teachers	Elementary and secondary	United States	Survey	Yes	Self-developed questionnaire	Academic performance
68. Tollefson et al., 1990	44 teachers	Elementary, secondary, and post-secondary	United States	Survey	Yes	Self-developed questionnaire	Academic performance
69. Tom and Cooper, 1986	25 teachers	Elementary	United States	Survey	Yes	Self-developed questionnaire	Academic performance
70. Upadyaya et al., 2012	16 teachers and 69 students	Kindergarten	Finland	Survey	No (two assessments for students, two assessments for teachers)	Self-developed questionnaire	Academic performance
71. Vernberg and Medway, 1981	30 teachers and 30 parents	Elementary	United States	Interview	Yes	Self-developed interview questions	Student misbehaviors
72. Vlachou et al., 2014	265 teachers	Elementary	Greece	Survey	Yes	Clark, 1997	Academic performance
73. Wang et al., 2015	523 teachers	Elementary and Secondary	Canada	Survey	Yes	McAuley et al., 1992	Occupational stress
74. Wiley and Eskilson, 1978	126 teachers	Elementary	United States	Experiment	Yes	Self-developed questionnaire	Academic performance
75. Wissink and de Haan, 2013	5 teacher and 54 parents/caregivers	Elementary	Netherlands	Content analysis	Yes	Conversations between teachers and parents; Graham, 1991	Academic performance
76. Woodcock and Jiang, 2013	103 teachers	Elementary and secondary	China	Survey	Yes	Questionnaire adapted from Clark (1997) and Woodcock and Vialle (2011)	Academic performance
77. Woolfson and Brady, 2009	99 teachers	Elementary	United Kingdom	Survey	Yes	Questionnaire adapted from Clark (1997)	Academic performance
78. Woolfson et al., 2007	199 teachers	Elementary	United Kingdom	Survey	Yes	Brady and Woolfson, 2008	Academic performance
79. Yehudah, 2002	51 teachers and 51 students	Secondary	Nigeria	Survey	Yes	Students’ oral essays followed by self-developed questions	Academic performance

**Table 3:** Overview of study outcomes, domains, and dimensions.

Attribution dimensions	Types of attributions	Focus of the papers	Number of studies	Median sample size (range)
Interpersonal	Academic performance	Prevalence	22	48 (8–390)
		Teacher outcome	10	82 (8–277)
		Responsibility	9	39 (8–202)
		Student outcomes	3	16 (16–53)
		Moderating factors	17	102 (1–390)
		Disagreement	3	7 (4–8)
Interpersonal	Classroom misbehaviors	Prevalence	19	108 (13–473)
		Teacher/student outcome	5	110 (13–391)
		Moderating factors	9	223 (73–473)
		Disagreement	4	54 (23–214)
Intrapersonal	Occupational stress	Prevalence	6	505 (222–687)
		Teacher outcome	4	505 (416–614)

## Results

### Interpersonal Attributions: Student Performance

Most research on teachers’ causal attributions has explored teachers’ beliefs concerning the causes underlying their students’ academic performance with respect to *success* and *failure* outcomes. However, it should be noted that teachers’ perceptions of student performance are multifaceted and subjective in nature, with some studies examining teacher-perceived student success or failure as pertaining specifically to academic marks (e.g., Wiley and Eskilson, 1978; Butler, 1994) or student competition (e.g., top performers; Beckman, 1976), and others focusing instead on student improvement or learning gains as a successful outcome (Emmerich et al., 2006; Espinoza et al., 2014). In many studies, although teachers are directly asked to reflect on a particular student success or failure scenario, they are typically provided with no specific guidance by researchers as to the standard against which determinations of success and failure are to be made (e.g., Wiley and Eskilson, 1978; Cooper and Burger, 1980; Fennema et al., 1990; Clark, 1997). Accordingly, in the present review we operationalize the terms success and failure in a more general manner such that success concerns satisfactory academic work as perceived by teachers, with student failure being perceived as generally unsatisfactory academic work.

#### Attribution Prevalence

Findings from Burger et al. (1982) suggest that teachers tend to attribute students’ academic performance to factors that are internal to the student and stable over time, particularly when the outcome matches teachers’ original expectations toward the students (ability, consistent effort, student personality). Conversely, teachers’ instead tend to implicate unstable factors when explaining student performance that was not consistent with teachers’ initial expectations (instructional methods, temporary effort). It is important to note in the study by Burger et al. (1982) that teachers’ expectations were not assessed as a cognitive consequences of attributions to stable/unstable factors as proposed in Weiner’s theory (expectations for future success), but rather as in indicator of teachers’ satisfaction with their students’ performance (having satisfied past expectations for student success). Teachers have also been found to be more likely to explain student failure as due to factors internal to the student (e.g., student ability, effort) or family influences than teacher- and school-related issues (Burger et al., 1982; for similar findings see Rolison and Medway, 1985; Hall et al., 1989; Tollefson et al., 1990; Jager and Denessen, 2015).

More specifically, approximately half of the teachers in Tollefson et al.’s (1990) study attributed student failure to student effort, with a significant proportion of attributions also involving external family variables (30%) and other internal student characteristics (77%; e.g., previous experiences, emotional maturity, interest, attention), However, only 2% of teachers in this study attributed student failure to teacher characteristics, and no attributions were reported to task difficulty. Results from Jager and Denessen (2015) similarly showed teachers to primarily explain students’ failure as due to internal student factors (e.g., attention, motivation, effort) than lesson difficulty or instructional quality (least common), reporting greater blame when failure was perceived as internal to and controllable by the student and lower criticism following external and uncontrollable attributions. Although other study findings suggest that teachers’ may focus especially on students’ external and uncontrollable antecedents to explain student performance (e.g., influence of prior teachers, learning experiences, or other students; Bar-Tal and Guttmann, 1981; Rolison and Medway, 1985; Hall et al., 1989; Bertrand and Marsh, 2015), existing findings generally suggest that teachers are more likely to attribute student failure to factors internal to the students themselves.

In addition to studies exploring teacher attributions for student failures, studies comparing their attributions for student success have also been conducted. Overall, findings suggest that whereas teachers tend to attribute student failure to factors that are internal to students, and external to themselves, they are most likely to attribute student success to themselves (Guskey, 1982; Yehudah, 2002). For example, whereas teachers might tend to attribute student failures to students’ lack of effort, they are instead found to attribute students’ success to their instructional strategies (Gosling, 1994; Kulinna, 2007). However, other studies suggest that although teachers may show a tendency to take responsibility for student success (e.g., instructional quality), they nevertheless give credit to students for their success (e.g., student ability, effort; Ross et al., 1974) as opposed to external factors such as luck (Natale et al., 2009).

In addition to the types of attributions made by teachers for student performance in general, teachers’ attributions nonetheless differ depending on the student. As outlined in early research on self-fulfilling prophecies (Jussim and Harber, 2005; Jussim et al., 2009), teachers often assign “labels” to their students to differentiate their potential for success (e.g., perceived ability level), with their attributions for subsequent student performance tending to correspond with those pre-existing labels (Rejeski and McCook, 1980; Reyna, 2000, 2008). For example, although a “high-ability” students’ success would likely be attributed to ability, a success outcome for a “low-ability” student would instead be more often attributed to the effort or luck. Conversely, teachers have been found to report more external or unstable attributions when failure is experienced by a “high-ability” student (e.g., task difficulty, temporary low effort), with failure by “low-ability” students more commonly attributed to low aptitude (Cooper and Burger, 1980; Rejeski and McCook, 1980; Fennema et al., 1990).

Research specifically exploring variability in teachers’ attributions for student performance further demonstrates both consistency, in terms of predictable attributional tendencies, as well as variability from one student to the next (e.g., Jager and Denessen, 2015). More specifically, findings indicate that causal attributions to factors such as student ability, family, class difficulty, and instructional quality are consistently reported by teachers as responsible for students’ poor academic performance. Teachers have also been found to consistently attribute student failure as due to low motivation, low ability, and family difficulties as opposed to teaching-related factors (e.g., lesson difficulty, instructional quality; Jager and Denessen, 2015). However, some attributions have been found to vary significantly depending on the student, such as attributions for student failure to student attention and effort (Jager and Denessen, 2015). Nevertheless, as related findings suggest that teachers tend to perceive student effort as a stable internal trait (e.g., laziness; Tollefson et al., 1990), failing students believed to be investing insufficient effort may nevertheless not be expected to improve.

#### Relations With Teacher Emotions and Behavior

According to Weiner’s (1985, 2000, 2010) attribution theory, the way in which teachers perceive the causes of their students’ performance can affect teachers’ emotions that, in turn, predict their teaching behaviors. Conversely, this theory thus also suggests that the behaviors and emotions conveyed by teachers can be interpreted by students as conveying teachers’ underlying attributional beliefs concerning their students’ performance (Frenzel and Stephens, 2013). Studies suggest that teachers tend to show more anger and disappointment when attributing student failure to low effort, and more empathy, pity, and helplessness to students perceived as low in ability (Butler, 1994; Georgiou et al., 2002a; Woolfson and Brady, 2009). Findings further indicate that teachers’ expression of sympathy following student failure can negatively impact student motivation and performance by implying attributions to low ability, with teachers’ expression of anger instead indirectly contributing to performance improvements by fostering students’ beliefs in their ability to succeed (Graham, 1984a).

In addition to emotions publically expressed by teachers, findings also show teachers’ attributions concerning student achievement to impact their personal emotional experiences. For example, a study by Prawat et al. (1983) with United States primary school teachers found teachers to experience more pride and gratitude when a student perceived as high in effort succeeded as well as higher levels of guilt when a high-ability student failed. In addition, study findings suggested that teachers experience a range of emotions when they perceive a significant change in student effort. More specifically, whereas teachers reported higher levels of pride and happiness when a low-ability student substantially increased their study efforts, they reported feeling more guilt and less gratitude following a perceived drop in effort in high-ability students. Results similarly showed teachers to experience surprise following unanticipated changes in student performance, such as when a high-ability student failed or low-ability student succeeded. These results suggest that teacher emotions are indeed linked with their causal attributions for their students’ performance, likely due to teachers perceiving personal responsibility for the change, and particularly so when changes in students’ study habits or achievement levels are unexpected.

Teachers’ attributions for student performance have also been found to impact teachers’ instructional behaviors. Findings show attributions for poor student performance to low ability to correspond with encouragement of students and willingness to provide assistance, with attributions for student failure to low effort generating more criticism toward students, negative feedback, and a lower tendency to offer help (e.g., suggestions; Cooper and Lowe, 1977; Tollefson and Chen, 1988; Butler, 1994; Matteucci et al., 2008b; Woodcock and Jiang, 2013). Teachers also tended to call on other students to assist and offer expedited help (e.g., providing correct answers) to students perceived as low in ability and responded more strictly to students perceived as low in effort in requiring them to invest more time on future tasks (Butler, 1994). However, a study by Rodriquez and Tollefson (1987) with teachers in Costa Rica found opposite results, with teachers who perceived students as failing due to insufficient effort reporting a greater willingness to help and more positive perceptions of the student (e.g., liking, expected success). Nevertheless, the reasons for these contradictory findings were unclear due to the underlying perceived dimensionality of low effort in Rodriquez and Tollefson’s (1987) study being unknown and the possibility of cultural differences concerning the perceived controllability of low effort.

Studies have also examined the effects of causal attributions on instructional responses to students’ failures with respect to their underlying causal dimensions. Research by Reyna and Weiner (2001) showed that teachers who made attributions for students’ failures to factors they perceived as personally controllable by the student (e.g., laziness, temporary lack of effort) provided not only negative feedback but endorsed retributive punishment goals. In contrast, teachers who believed student failure to result from uncontrollable factors (e.g., transfer student, low ability) or unstable factors (e.g., a temporary decrease in student effort) were found to report more utilitarian punishment goals. Moreover, teachers who believed student failure to be due to factors that were both controllable *and* stable in nature, such as persistent student laziness, were those most likely to use retributive punishment goals (Reyna and Weiner, 2001, study 2).

#### Perceptions of Responsibility

An additional important component of the interpersonal aspect of attribution theory is the extent to which individuals perceive others as personally responsible for their experiences. Although perceptions of responsibility are not proposed in Weiner’s model as a subcomponent of, or replacement for, causal attributions, they are nevertheless clearly identified as a proximal critical cognitive consequence of the types of causal attributions teachers that, in turn, influence subsequent emotions and behaviors. As such, whereas the concept of perceived responsibility is not identical to, nor interchangeable with, the preceding causal attributions in Weiner’s theory, they nevertheless are assumed to strongly reflect the types of attributions being made (concerning perceived controllability) and have thus been repeatedly examined as a proxy for teachers’ causal attributions in previous research. Existing research in educational settings has indeed long suggested that when students are held responsible for their failure experiences, they are more likely to be perceived as deserving of punishment as opposed to rewards (Weiner, 2000, 2001). Findings with teachers have since expanded on these findings in showing that when students’ failures are perceived as due to lack of effort rather than lack of ability, teachers are not only more likely to experience greater anger but also believe students to be more responsible for their failure outcome (Matteucci, 2007; also see Matteucci et al., 2008a for a review).

Additionally, findings from Matteucci (2007) show higher levels of perceived student responsibility for failure to lead to retribution-motivated punishment behaviors such as assigning lower marks or more homework. Results further suggested that when student failure was interpreted by teachers as due to lack of ability, teachers who perceived the student to be less personally responsible were more likely to adopt a utilitarian instructional response (e.g., assistance, encouragement). With respect to mediation effects, Reyna and Weiner (2001) further observed that teachers’ attributions to factors controllable by the student on teacher emotions (anger, sympathy) were mediated by perceived student responsibility, with the positive relationship between teachers’ perceptions of student responsibility and retributional punishment goals further mediated by teachers’ emotions (i.e., lower sympathy). Concerning teachers taking personal responsibility for student failures, findings are mixed. Whereas some showing teachers to perceive themselves as less responsible than students for student failures (e.g., regardless of how it is attributed by teachers; Tollefson et al., 1990, 1991; Matteucci, 2007) and others showing teachers to take more responsibility for students’ failures than their successes (e.g., Ross et al., 1974; Cooper and Lowe, 1977; Cooper and Burger, 1980; Burger et al., 1982; Tom and Cooper, 1986).

#### Relations With Student Outcomes

In addition to research examining empirical links between teachers’ causal attributions for student performance and their own emotions and instructional behaviors, studies have further explored how teachers’ attributions impact their students’ motivation and performance. For example, Natale et al. (2009) found that when teachers made more effort and ability attributions for student success, their students reported higher motivation levels and better performance on reading-related tasks. More interestingly, students’ academic motivation and performance was found, in turn, to influence their teachers’ attributions: The higher a student’s motivation and performance, the stronger the tendency for teachers to attribute the student’s success to high effort and ability. However, when teachers attributed the student’s success to low task difficulty or having provided assistance, lower levels of reading-related motivation and poorer performance were subsequently observed.

Results from a study with kindergarten students and their teachers in Finland further suggested that teachers’ causal attributions can influence student interest in mathematics (Upadyaya et al., 2012). More specifically, this study showed that the greater students’ intrinsic value in mathematics, the more likely their teachers were to attribute math success to factors internal to the student, such as ability and effort, as opposed to external factors such as task easiness. Such internal attributions in turn, contributed to even higher subsequent levels of student intrinsic value and academic performance in mathematics. Similar results were observed in a study with teachers in Estonia (Tõeväli and Kikas, 2016) in which higher levels of student math performance corresponded with a lower likelihood of teachers attributing students’ success in math to teacher assistance and a greater likelihood of attributing it instead to student ability. Nevertheless, study findings also showed that students tended to perform poorly in math when their teachers attributed students’ math successes as due to teacher assistance, with students also found to perform more poorly when teachers attributed students’ math failures to low ability. In other words, poor performance tended to result in teacher attributions to low student ability that, in turn, contributed to even poorer grades thus perpetuating a detrimental attribution-achievement cycle (Tõeväli and Kikas, 2016).

Finally, teachers’ causal attributions for student performance have been found to impact students’ perceptions of their peers by way of teachers’ attributional cues (e.g., verbal communication, emotional expression, body gestures). In one experimental study, students were asked to watch videotapes in which a teacher acted so as to convey attributional cues suggesting either low or high levels of student effort to two students, each of whom had scored 80% correct on a recent exam. Whereas the teacher praised the first student and encouraged them to continue their successful efforts, the second student was criticized for not having invested sufficient effort to perform better. Study findings showed students to not only perceive the first student as smarter (c.f., incremental self-theories of intelligence in which increased ability is assumed to follows from increased effort; see Dweck, 2013), but also indicate a greater preference for the student a study partner (Lord et al., 1990).

#### Moderating Teacher Variables

With respect to teacher characteristics found to moderate relations between their causal attributions for student achievement and classroom outcomes, findings suggest that teachers with greater *teaching experience* may be more likely than novice teachers to believe students’ successes or failures to be due to external, uncontrollable factors such as family background or socio-economic status (e.g., parental income, education; Georgiou, 2008). These teachers reported a stronger belief in student achievement as due to uncontrollable factors that are also stable over time, such as biological predispositions (e.g., innate intelligence), thereby de-emphasizing the teachers’ role in, and responsibility for, student achievement relative to novice teachers. In contrast, novice teachers tended to perceive themselves as having a greater impact on their students’ performance and reported stronger beliefs than more experienced teachers in the role of student effort and diligence in student success (Georgiou, 2008). However, contradictory findings were reported by Pirrone (2012) who found although teachers with over 30 years of experience did report a stronger tendency to attribute student failure to student immaturity (internal, uncontrollable), teachers with under 10 years of experience were instead more likely to blame parents or family for student failure (external attributions).

Differences in attributional patterns have also been observed for teachers based on their *level of education*, with findings showing teachers with university degrees to have a stronger tendency to attribute student failure to factors internal to and controllable by the student, such as lack of interest or motivation to learn, than teachers with a high school degree (Pirrone, 2012). Mixed results have also been reported concerning teachers’ *level of instruction* as a moderating factor. In early work by Guskey (1982), teachers who taught higher grade levels reported a stronger tendency to attribute student failure to task difficulty, with teachers of lower grade levels instead being more likely to attribute student failure to teachers’ own teaching ability and effort. In contrast, early findings from Hall et al. (1989) showed elementary and junior-high teachers to be more likely than senior-high teachers to attribute student failure both to causes internal to students (e.g., effort, ability, concentration, habits) or to external factors (e.g., task difficulty, home life). More recently, although findings do suggest that junior-high teachers may perceive themselves as more responsible than senior-high teachers when student failures are attributed to lack of student ability, the degree of perceived personal responsibility reported by both types of teachers was still significantly lower than that attributed to students (Matteucci and Gosling, 2004).

Existing research further indicates that teachers’ causal attributions may differ as a function of *learning disabilities instruction.* For example, Woolfson et al. (2007) found teachers of learning-challenged students at specialized institutions to perceive their students as having more personal control over their academic performance than teachers at mainstream schools who instead tended to make more uncontrollable, stable attributions for student difficulties. Results with British teachers showed teachers with more special education experience to also make more student-external attributions for learning difficulties (e.g., bad luck; Brady and Woolfson, 2008), with another study conducted with teachers in Greece further showing mainstream teachers to be more likely than special education teachers to attribute student failure to student persistent low effort (internal, stable; Vlachou et al., 2014). Finally, studies have examined *cultural differences* in teachers’ causal attributions, with scattered results suggesting that United States teachers make more effort-related attributions for student performance than German teachers (Kurtz et al., 1990), and that teachers in France perceive themselves as more responsible for student failure than their Italian counterparts (Matteucci and Gosling, 2004).

#### Moderating Student Variables

The impact of *student gender* on teacher attributions has consistently been explored in prior research (see Reyna, 2000 for a review). In addition to teachers tending to perceive boys as having higher math ability and girls as more capable with language topics (Georgiou, 2008), teachers additionally tend to attribute girls’ math success to effort and boys’ math success to ability (Fennema et al., 1990; Espinoza et al., 2014). Conversely, findings suggest that teachers are also more likely to attribute poor math performance for females to lack of ability, and for males to lack of effort. Although interventions to counter teachers’ attributional gender biases do show benefits, observed gains tend to be temporary with findings showing biased attributions reported by teachers to return to baseline levels within a year (Espinoza et al., 2014).

Studies also show teachers’ attributions to be influenced by *student ethnicity* (see Reyna, 2000, 2008 for reviews concerning teacher stereotypes concerning minority students). Specifically, a Netherlands study found teachers to be more likely to attribute satisfactory academic performance to effort for ethnic minority student (vs. ethnic majority students; Wissink and de Haan, 2013), with Canadian research further showing teachers to place greater emphasis on external factors (i.e., family background, socio-economic status) when evaluating the performance of aboriginal students (vs. non-aboriginal student; Riley and Ungerleider, 2012). Studies with United States teachers similarly show teachers to make fewer personally controllable and more external attributions for the performance of African-American students (e.g., family, social environment; Wiley and Eskilson, 1978), with the performance of Caucasian students being more often attributed to student ability, effort, and other personal characteristics (Wiley and Eskilson, 1978; Tom and Cooper, 1986).

Finally, findings indicate that teachers’ attributions for student achievement are likely to differ depending on *student learning disabilities* (Conway, 1989; Vlachou et al., 2014). Results suggest that for students with learning difficulties, teachers are likely to attribute poor performance more to factors internal to the student, especially to limited ability, followed by low student effort or motivation (Vlachou et al., 2014). In contrast, teachers are less likely to attribute poor performance among students with learning disabilities to teaching methods (e.g., classroom management, curriculum) or student-external factors (e.g., family background) as compared to mainstream students (Conway, 1989). Conversely, findings show teachers tend to attribute academic success for learning-challenged students to low task difficulty or high student effort (Algozzine and Stoller, 1981).

#### Attributional Disagreement

As implied by the preceding findings, it is possible for teachers and students to differ on not only if an achievement outcome is interpreted as a success or failure, but also on the appropriate causal attribution for a given outcome. Studies suggest that teachers are indeed less certain than students when evaluating if a student’s performance constitutes a failure outcome (Juvonen, 1988), with both students and their parents tending to hold higher performance expectations than their teachers (Bar-Tal and Guttmann, 1981). For example, Juvonen (1988) found teachers’ and students’ attributions for math performance to differ in one third of cases (i.e., 28 of 84) with teachers attributing perceived failures to factors that persisted over time, such as low ability and laziness, and students instead being more inclined to make unstable failure attributions, such as fatigue or sloppiness. Similarly, interviews by Klassen and Lynch (2007) with students with learning disabilities and their teachers showed whereas students predominantly attributed their academic failures to unstable and controllable factors (i.e., lack of effort), most of the teachers interviewed attributed students’ failure to internal, uncontrollable, and stable factors (i.e., lack of ability). Concerning student success, findings from Juvonen (1988) again showed disagreement between the attributions reported by students and teachers, with teachers demonstrating a stronger tendency to attribute students’ success to stable factors, such as interest in math, and students instead reporting more attributions to unstable or external factors, such test-taking mood or support from parents.

### Interpersonal Attributions: Student Misbehavior

#### Attribution Prevalence

In addition to research examining teachers’ causal attributions for student achievement, studies have further explored how teachers attribute students’ behavior in class, particularly with respect to perceived misbehavior. Early work by Medway (1979) comparing teachers’ attributions for students’ learning difficulties (e.g., reading, understanding) and behavioral challenges (e.g., aggression, withdrawal) found that teachers primarily attributed learning problems to ability-related factors, while attributing behavioral problems to peer and parental factors (see also Arcia et al., 2000). Findings have additionally shown that whereas severity of student learning difficulties was positively correlated with teachers’ attributions to student ability (e.g., intelligence), severity of student behavioral problems instead corresponded to more personality attributions (Medway, 1979). Other studies have similarly revealed that whereas ability and effort tend to be the most commonly cited reasons for student learning problems, personality and parental factors are more often endorsed as reasons for behavioral difficulties (e.g., Medway, 1979; Hughes et al., 1993; Savina et al., 2014).

Despite these differences, findings concerning teachers’ attributional tendencies for student misbehavior are largely similar to those observed regarding attributions for low achievement. When faced with student behavioral problems (e.g., disobedience, disruption, aggression; mild, moderate, severe), teachers’ attributions tend to not implicate themselves (e.g., instructional method, curriculum, teacher attitudes, discipline) or administrative factors (e.g., teaching demands, class size) but instead consistently cite factors internal to the student (e.g., ability, effort, personality, social/physical skills) and parental variables (e.g., dysfunction, attitudes, interest; Vernberg and Medway, 1981; Christenson et al., 1983; Miller, 1995; Bibou-Nakou et al., 2000; Mavropoulou and Padeliadu, 2002; Ho, 2004; Kulinna, 2007; McAuliffe et al., 2009; Andreou and Rapti, 2010; Ding et al., 2010; Kauppi and Porhola, 2012; Savina et al., 2014). For example, whereas more than half of the teachers in Ding et al.’s (2010) study attributed students’ misbehaviors to students’ lack of effort, with substantial proportions citing various other factors internal to students (e.g., laziness: 32%, study habits: 23%, low interest: 20%), only 0.8% of teachers attributed students’ behavioral difficulties to their own instructional effectiveness or classroom management skills.

Similarly, Kleftaras and Didaskalou (2006) found teachers to primarily attribute student behavioral and emotional problems to family issues (74%) or student-related factors (17%; e.g., biological or psychological deficits), with only 6% of teachers reporting school context or teaching practices as important contributors. A study by Savina et al. (2014) that specifically examined teachers’ attributions for students’ externalizing behaviors (e.g., disruption, aggression) also showed problematic parental and peer relationships to be most often cited by teachers as critical determinants of students’ overt classroom misbehavior. However, although teachers have consistently been found to perceive the causes of student misbehavior as beyond their personal control and scope of responsibility (e.g., Bibou-Nakou et al., 2000; for atypical contradictory results, see Poulou and Norwich, 2000), research by Hughes et al. (1993) suggests that teachers may nonetheless perceive themselves as responsible for the success of their subsequent in-class behavior management efforts. Additionally, whereas parental factors are often cited by teachers as responsible for students’ classroom misbehavior (e.g., Soodak and Podell, 1994), other research suggests that teachers do not necessarily perceive parents as helpful for managing student behavior (Miller, 1995; also see Wiley et al., 2012 for a review on parents’ and teachers’ attributions concerning misbehaviors).

#### Relations With Teacher and Student Outcomes

Findings suggest that when teachers attribute student aggression to factors that are internal to students and stable over time, they also tend to report more maladaptive teaching behaviors (e.g., expressing anger, sarcasm) that, in turn, lead to greater peer dislike of the student (McAuliffe et al., 2009). Additionally, when teachers believed that students’ behavioral problems as due to student intelligence or other factors perceived as beyond the teachers’ own control, they also reported a stronger tendency to seek professional consultation or special education services (Soodak and Podell, 1994). Concerning teachers’ attributions for student disruptive behavior to factors external to themselves (e.g., students’ family-related issues, student internal factors), findings suggest that such attributions may reflect lower levels of teacher motivation (i.e., self-efficacy) in dealing with behavioral difficulties as well as greater efforts to seek out parental assistance (Soodak and Podell, 1994). Conversely, teachers who attribute student misbehavior to teaching- or class-related reasons have been found to report not only greater sympathy for and confidence in students with emotional and behavioral difficulties but also a greater willingness to improve their teaching strategies (Soodak and Podell, 1994) as well as to personally intervene and provide assistance (Hughes et al., 1993; Poulou and Norwich, 2000). Research further suggests that whereas teachers’ attributions for student misbehavior to factors internal to students tend to have mixed relations with teacher burnout indices (i.e., higher levels of both personal accomplishment and emotional exhaustion), attributions to contextual factors (e.g., classroom environment) are associated with lower teacher burnout (i.e., lower depersonalization; Bibou-Nakou et al., 1999).

#### Moderating Variables

Similar to research on teachers’ attributions for student performance, findings indicate that greater *teaching experience* tends to correspond with a greater likelihood of teachers perceiving students and family-related factors as more important contributors to students’ behavioral problems than school- or teacher-related issues (Mavropoulou and Padeliadu, 2002). However, other research more specifically suggests that older teachers (>47 years) as well as younger teachers (<35 years) are more likely to endorse parenting-related attributions for students’ emotional and behavioral problems than middle-aged teachers, with this intermediate group assumed to hold differing attributional views due to having more experience than younger teachers yet holding less conservative views toward instruction than older teachers (Kleftaras and Didaskalou, 2006). With respect to the moderating role of *level of instruction*, senior high teachers have been found to demonstrate a stronger tendency to attribute student misbehavior to non-academic factors beyond their personal control than junior high and elementary school teachers (e.g., parental skills, community environment; Kulinna, 2007). In addition, elementary school teachers were found to be more likely to attribute students’ misbehaviors to poor learning habits, whereas middle and high school teachers tended to attribute students’ misbehaviors to low effort (Ding et al., 2010).

Multiple studies have also examined *cultural differences* as potential moderators of teachers’ attributions for behavioral difficulties. For example, research by Ho (2004) found that whereas Australian teachers focused on student ability, Chinese teachers instead placed greater emphasis on family issues. Findings from Gibbs and Gardiner (2008) further suggested that British teachers were more likely than Irish teachers to attribute challenging student behaviors to factors external to the student such as teacher behavior, parental involvement, or class curriculum. As for *student ethnicity*, results from Jackson (2002) indicated that teachers more frequently provided situational explanations (e.g., other people) for the misbehavior of Caucasian students, whereas internal explanations (e.g., student personal characteristics) were more often reported for non-Caucasian American students. Finally, studies have also found *student gender* to moderate teachers’ attributions, with mixed results suggesting that teachers may be more likely to attribute female students’ misbehavior to internal and uncontrollable factors (e.g., personality characteristics; Savina et al., 2014), or more inclined to attribute male students’ behavioral problems to unintentional (i.e., uncontrollable) factors (Arbeau and Coplan, 2007).

#### Attributional Disagreement

Multiple studies comparing teachers’ and their students’ causal attributions concerning students’ classroom difficulties have observed disagreement between each set of reports. For example, in an interview study with secondary school students and their physical education teachers in the United States, it was found that teachers were more likely than their students to attribute misbehavior to stable factors, such as family-related reasons, whereas students tended to report attributions to unstable factors, such as attention-seeking or boredom (Cothran et al., 2009). In a vignette study by Guttmann (1982), whereas students tended to attribute another student’s misbehavior to contextual features (e.g., teacher attitudes, peer influence), teachers were more likely to attribute misbehavior to factors internal to the student (e.g., students’ psychological problems, need for teacher’s attention). Comparable findings were found in a study by Hui (2001) comparing Hong Kong students’ and teachers’ causal attributions toward students’ classroom difficulties, showing students’ to be more likely to attribute their problems to teacher- or school-related factors (e.g., teacher bias, school rules), and teachers instead tending to blame students, peers, or parents for students’ difficulties. Other findings have further shown teachers to be more likely than students’ parents to not only rate behavioral problems as serious in nature but also as resulting from underlying biological factors (Savina et al., 2014).

### Intrapersonal Attributions: Occupational Stress

#### Attribution Prevalence

Beyond studies exploring the prevalence, correlates, and potential moderators of teachers’ *interpersonal* attributions for student outcomes, limited research has also explored the implications of teachers’ *intrapersonal* attributions for their own teaching-related stress. Research on teachers’ causal attributions for their occupational stress found that teachers most frequently attributed their stress to external and uncontrollable factors and were least likely to report occupational stress as due to internal reasons (Wang et al., 2015). More specifically, studies suggest that teachers primarily attribute their occupational stress to administrative factors (e.g., excessive workload, lack of respect, lack of time), structural issues (e.g., high student–teacher ratio; McCormick and Solman, 1992a,b; McCormick, 1997; Manassero et al., 2006), student reasons (e.g., low student motivation), and were less likely to attribute stress to their own classroom management or instructional skills (Manassero et al., 2006). Finally, cross-cultural research by McCormick and Shi (1999) suggests that although both Australian and Chinese teachers make primarily external attributions for teaching-related stress, Australian teachers tend to focus on bureaucratic factors (e.g., government) whereas Chinese teachers tended to blame societal issues.

#### Relations With Teacher Outcomes

In a study by Manassero et al. (2006), teachers’ causal attributions for personal stress were investigated with respect to three attributional dimensions outlined in Weiner’s (1985) attribution theory (locus, stability, controllability) and two additional dimensions derived from earlier theoretical formulations: *intentionality* and *globality*. Whereas *intentionality* refers to the conscious, deliberate nature of a personally controllable attribution (e.g., forethought as to potential consequences), *globality* refers to the situational scope of a given attribution (i.e., domain-general vs. context-specific; Abramson et al., 1978). Study results showed teachers who made more stable, global, or intentional attributions for their teaching-related stress to also report higher levels of burnout (emotional exhaustion, depersonalization). Further, although internal or uncontrollable attributions corresponded with greater burnout (exhaustion and depersonalization, respectively), teachers who reported more unstable, controllable, or specific (not global) attributions for teaching stress reported higher levels of personal accomplishment.

Furthermore, findings also show significant relations between teachers’ attributions for teaching-related stress and psychosocial outcomes beyond occupational burnout. For example, a study with Canadian teachers by Wang et al. (2015) showed that although attributions for teaching stress to internal or personally controllable causes did correlate with higher job satisfaction, attributions to personally controllable factors further corresponded to lower emotional exhaustion, fewer illness symptoms, and lower quitting intentions. In contrast, teachers who attributed their occupational stress to stable reasons, or factors under the control of others, tended to reported poorer well-being outcomes (e.g., burnout, job satisfaction, illness, quitting intentions). Comparable findings were also observed by McCormick and Barnett (2011) who found that teachers who blamed students for their occupational stress reported higher depersonalization and emotional exhaustion, whereas teachers who blamed themselves or their students also tended to have lower levels of personal accomplishment. Results were also found with the dimension locus of causality, with teachers who blamed external factors involving government or educational policies for their occupational stress reporting higher emotional exhaustion. Moreover, whereas teachers who reported external factors as primarily responsible for their occupational stress reported lower job satisfaction and greater burnout, particularly those who implicated students as their primary source of stress (e.g., lack of administrative support; McCormick, 1997; McCormick and Barnett, 2011), teachers who instead reported a willingness to take personal responsibility for their shortcomings reported higher job satisfaction (McCormick, 1997).

## Discussion

As evidenced by the scope of studies reviewed, there exists a long and international history of empirical research on the prevalence and consequences of teachers’ causal attributions for varied instructional challenges. With respect to findings applying an interpersonal approach to Weiner’s (2000) attribution theory, existing research has principally explored the types and correlates of attributions made by teachers for students’ academic performance and misbehavior. In contrast, research on teachers’ intrapersonal attributions for their own occupational stress is limited yet encouraging in showing significant relations with teacher well-being. Consistent with attribution theory, empirical findings indeed showed teachers’ causal attributions to correspond with not only their teaching-related emotions and instructional behaviors, but also observed outcomes in students, with these relations having been further explored with respect to moderating student and teacher variables. Overall, Weiner’s attribution theory has for decades provided a useful, foundational framework within which to examine the implications of teachers’ causal attributions for personal and instructional outcomes, with the pattern of results that emerge across these studies suggesting consistent themes as outlined below.

### Prevalence of Teachers’ Attributions: Common Themes

#### Fundamental Attribution Error

In social psychology research, it has been argued that individuals tend to interpret events in stereotyped ways and as such are motivated to overemphasize the role of dispositions in causal thinking about others (Ross, 1977). Referred to as the *fundamental attribution error*, this bias involves “the tendency for attributors to underestimate the impact of situational factors and to overestimate the role of dispositional factors in controlling behavior” (Ross, 1977; p. 183). Moreover, this attributional hypothesis further asserts that the “actors” and “observers” of an event consistently differ in the types of attributions they select, with the actors having a greater tendency to attribute behaviors to situational forces or constraints, and observers instead being more inclined to make attributions implicating the actor’s abilities, dispositions, and attitudes (also referred to as “actor–observer bias”; Jones and Nisbett, 1971). By way of explanation, it is commonly asserted that such interpretation patterns serve to enhance self-esteem, minimize negative affect, and also defend against ego threats and compromised perceptions of one’s abilities (Heider, 1958; Jones and Davis, 1965; Ross, 1977).

As outlined above, the fundamental attribution error is clearly evident in research examining the types of attributions made by teachers for student performance, misbehavior, and teaching stress. Moreover, the present review underscores the double-edged nature of biased attributions in showing teachers to not only report self-protective attributions in failure situations but also self-enhancing attributions following success (hedonic bias; Miller and Ross, 1975). Across multiple empirical investigations, teachers have consistently been found to attribute student difficulties (i.e., poor academic performance, behavioral difficulties) to student-related or family-related reasons, and much less frequently report perceiving the problem as teaching-related. Further, teachers and students are often found to report different attributions for students’ academic challenges, with students tending to see their personal failures as temporary and controllable and teachers instead regarding such outcomes as more serious, persistent, and not controllable by students. In addition to teachers’ interpersonal attributions for student outcomes, this ego-defensive attributional pattern is also reflected in the attributions teachers make for their own occupational stress. As suggested by McCormick and Solman (1992a,b) and Wang et al. (2015), teachers also tend to attribute teaching-related stress to factors external to themselves (e.g., students, society, government) presumably in an effort to mitigate negative emotions as well as the implication of teaching-related setbacks as personal failures.

However, critics contend that an ego-defensive explanation for this attributional pattern should be viewed with skepticism, and instead propose a simpler account for this attributional asymmetry. More specifically, it is postulated that personal successes are simply perceived as more likely to occur than failure due to positive self-perceptions requiring quick cognitive access to prior success experiences, with failure-related memories instead being more difficult to cognitively access. As such, whereas personal successes (e.g., a student succeeding) are more likely to be attributed to self-relevant factors (e.g., teaching strategies), personal failures (e.g., a student performing poorly) are less easily attributable to internal factors and thus more likely to be attributed to readily external variables (e.g., observable characteristics of the student). This tendency to attribute student failure to factors internal to the student is then further exacerbated by teachers, as external observers, being less aware than their students of the situational contributors to student failure (Miller and Ross, 1975).

Accordingly, further research is needed to examine teachers’ motivations underlying the types of attributions they select, for example, whether external attributions mitigate the effects of perceived ego threat on subsequent emotions or instead mediate the effects of limited situational awareness on subsequent instructional choices. Moreover, further research on factors that moderate this attributional bias is warranted given findings with teachers that did not demonstrate the fundamental attribution bias, but rather the opposite. For example, results from studies by Burger et al. (1982) showed teachers to in fact be more willing to assume responsibility for student failure than for their success. Findings from Rodriquez and Tollefson (1987) also showed teachers to be more willing to offer assistance to students to whom they attributed low effort rather than low ability. An earlier study by Ross et al. (1974) further showed teachers to rate student ability and aptitude as more important determinants of student success than student failure, and were more likely to attribute student failure as due to teacher ability, aptitude, and motivation.

#### Moderating Factors

Beyond overall patterns concerning the types of causal attributions made by teachers, studies have also consistently explored potential moderators of attribution prevalence – most notably with respect to *teaching experience*. More specifically, findings from multiple studies suggest that more experienced teachers tend to make more uncontrollable attributions for students’ academic failure (e.g., innate intelligence, family background; Georgiou, 2008) and misbehavior (e.g., poor parenting; Kleftaras and Didaskalou, 2006). As an explanation for this trend, Georgiou (2008) suggests that novice teachers may hold unrealistic beliefs in their ability to effectively intervene and improve their students’ performance through personal perseverance and encouraging students to invest greater effort. However, it is presumed that as teachers gain more experience they become more realistic and more comfortable with the belief that not all students can succeed due to various uncontrollable and stable factors that can significantly undermine their efforts (Pirrone, 2012). Alternatively, it has been asserted that because teachers with greater experience are more competent and more certain that their instructional strategies are sufficient for student success, they instead focus on factors unrelated to their instructional methods as responsible for student difficulties due to already having maximized the potential impact of their teaching methods (Georgiou, 2008). Additionally, findings suggest that the attributions made by teachers of students with special needs can differ depending on experience in that although teachers generally attribute the failure of special needs students to ability and success to effort (e.g., Algozzine and Stoller, 1981), teachers with more special education experience are more likely to see student failure as more controllable by the student (Woolfson et al., 2007) and acknowledge the role of factors external to the student (e.g., Brady and Woolfson, 2008).

Concerning the moderating effects of *level of instruction*, it has been suggested that as content difficulty increases, students’ academic performance and behavior are unlikely to be based on high effort alone due to the increasingly evident role of uncontrollable factors such as student intelligence, maturity, or family background. Accordingly, teachers at higher grade levels tend to feel less personally responsible for the performance and behavior of their students than teachers of lower grade levels (Mavropoulou and Padeliadu, 2002; Matteucci and Gosling, 2004). Furthermore, teachers were found to also make different attributions based on *student demographics*. More specifically, whereas teachers have been found to be more likely to attribute girls’ successes to high effort and failures to low ability, they instead tend to attribute boy’s successes to high ability and failures to low effort (e.g., Espinoza et al., 2014). Similarly, although a girl’s classroom misbehavior is likely to be attributed by teachers to internal and uncontrollable factors (e.g., personality), misbehavior by boys is more often viewed an unintentional (e.g., Arbeau and Coplan, 2007).

Moreover, teachers tend to believe the academic performance of *ethnic minority* students to be due to factors that are external to and uncontrollable by the students (e.g., family background), and instead tend to make student-internal attributions for the academic performance of ethnic majority students (e.g., ability, effort; Wiley and Eskilson, 1978; Riley and Ungerleider, 2012). Concerning student misbehaviors, however, teachers also shown a tendency to believe behavioral problems evidenced by ethnic minority students to be due to factors internal to the students (e.g., student characteristics, personality), with similar behavior exhibited by ethnic majority students’ instead attributed primarily to situational or external factors (e.g., influence of other people; Jackson, 2002). In sum, teachers’ causal attributions have been found to be significantly impacted by their experiences interacting with students as well as their existing knowledge, stereotypes, and perceptions concerning their students. Thus whereas overall findings concerning attribution prevalence suggests that teachers’ attributions may be systematically biased (e.g., fundamental attribution error), this pattern may be mitigated by specific teacher and student characteristics.

### Implications of Teachers’ Attributions for Instruction and Well-Being

#### Students and Instruction

With respect to the potential influences of teachers’ causal attributions on students, attribution theory suggests that student motivation and achievement can indeed be influenced by teachers’ causal attributions by way of attributional cues (e.g., emotional expression, body languages, verbal feedback; see Graham, 1984a,b, 1990; Weiner, 1985, 2000, 2010; Graham and Williams, 2009). Moreover, research on the *self-fulfilling prophecy* asserts that teachers’ inaccurate causal attributions concerning low ability can develop into persistent stereotypes that lead teachers to expect less of these students, interpret subsequent outcomes as conforming to this stereotype, and interact with these students in a manner that elicits further poor performance (Jussim and Harber, 2005; Jussim et al., 2009). Overall, there exists a substantial existing literature suggesting that teachers’ attributional beliefs concerning the causes of students’ classroom behavior and performance can significantly impact subsequent student outcomes (e.g., Cooper and Burger, 1980; Rejeski and McCook, 1980; Fennema et al., 1990; Lord et al., 1990).

In the current review, these theories were supported by findings showing teachers who attributed their students’ performance to low ability to report more sympathy and lower expectations for these students, as well as greater use of instructional techniques intended to punish as opposed to assist low-ability students (e.g., Butler, 1994; Georgiou et al., 2002a). Students perceived as low ability, in turn, were also not only more likely to attribute their failure to limited ability but report lower expectations for success in their studies (Graham, 1984a). Conversely, teachers who perceived student failure as due to low effort were found to experience greater anger toward these students and sustained or increased performance expectations (Butler, 1994; Georgiou et al., 2002a) and further preferred correctional methods that were utilitarian and informative in nature (Reyna and Weiner, 2001). Poor-performing students perceived as lacking in effort, in turn, perceived teachers’ anger as indicating that improvement was possible and reported more positive success expectations (Graham, 1984a). However, it is important to note that as prior empirical studies have focused specifically on teachers (e.g., self-report attributions, expectations, emotions) or students (e.g., self-report success expectations, perceived teacher attributions) in exploring the influences of teachers’ attributions and affective cues on students, research assessing both teachers and students simultaneously is lacking. Accordingly, the specific theoretical assumptions in Weiner’s (1985, 2000, 2010) theory concerning possible links between interpersonal and intrapersonal attributions have yet to be empirically examined.

#### Teacher Well-Being

Studies concerning the effects of attributions on teachers’ well-being have yielded mixed yet encouraging results. More specifically, internal attributions concerning teachers’ occupational stress have been found to have both negative effects (e.g., Manassero et al., 2006; McCormick and Barnett, 2011) and positive effects on well-being outcomes (e.g., job satisfaction; Wang et al., 2015), with external attributions for stress showing similarly mixed results (e.g., Bibou-Nakou et al., 1999; McCormick and Barnett, 2011). Nevertheless, findings on the effects of personally controllable attributions on well-being are notably consistent in showing teachers who focus on the controllable aspects of their occupational stress to report lower burnout, fewer illness symptoms, greater job satisfaction, and weaker intentions to quit (McCormick, 1997; Manassero et al., 2006; Wang et al., 2015). Moreover, it has been proposed that the conceptual overlap between personally controllable attributions and internal causal attributions may be responsible for the mixed effects of the latter in existing research. According to Wang et al. (2015), as attributions that are internal to an individual may be either controllable (e.g., effort) or uncontrollable in nature (e.g., ability), analyses that evaluate both internality and controllability simultaneously may show negative effects for internality due to multicollinearity. More specifically, studies in which personal controllability is evaluated alongside internality may in fact be statistically removing the beneficial elements of internality by controlling for personally controllable aspects (e.g., stress due to insufficient effort), leaving the uncontrollable elements of internality to be assessed (e.g., low ability as responsible for occupational stress).

### Future Directions

Results from the current review suggest that teachers’ causal attributions can influence their emotions and, in turn, instructional behaviors that have significant implications for students’ academic performance, motivation, and behaviors. However, whereas specific links between teachers’ attributions and their interactions with students have been empirically explored, notably few studies have examined the actual effects of teachers’ attributions on their students by examining both teacher and student data simultaneously. Additionally, in contrast to the majority of published studies on teacher attributions having focused primarily on the implications of attributions for students’ problems (performance, behaviors), little research has been conducted to date concerning the effects of teachers’ attributions on their personal well-being. Accordingly, further research is needed in which the correspondence between reports by both teachers and students is examined. Other than that, research is also needed concerning the influences of teachers’ attributions for occupational stressors and specific classroom experiences on their psychological and physical health to provide a more thorough examination of the implications of teachers’ causal attributions for not only their students’ but also teachers’ own personal development.

Another limitation of the research reviewed is the methodological overreliance on predetermined lists of specific teacher attributions, namely student ability and effort (e.g., Hall et al., 1989; Tollefson et al., 1990; Natale et al., 2009). Accordingly, future studies in which measures that more accurately capture Weiner’s (1985, 2001, 2010) attribution theory by measuring the underlying attributional dimensions are encouraged (e.g., stable vs. persistent lack of student effort, Reyna and Weiner, 2001; for further examples, see Manassero et al., 2006; Wang et al., 2015). Moreover, as studies have to date explored teacher attributions using inconsistent self-report measures (e.g., validated scales with general populations or teachers, self-designed measures, vignettes, interviews; McAuley et al., 1992; Reyna and Weiner, 2001; McCormick et al., 2006; Klassen and Lynch, 2007; Ding et al., 2010), greater research efforts to validate a more comprehensive measure to examine teachers’ attributions concerning both interpersonal challenges (e.g., student difficulties) and intrapersonal struggles (e.g., occupational stress) are warranted to broaden and systematize our understanding of how teachers interpret various classroom and occupational experiences. Similarly, the development and assessment of outcome measures that do not rely solely on self-report methods (questionnaires, interviews) are needed to more objectively examine the real-world impact of teacher attributions on instructional behavior and well-being (e.g., classroom observations, physiological measures; Schwerdtfeger et al., 2008).

With respect to additional methodological considerations, the present overreliance on self-report measures in existing work on teachers’ attributions also warrants consideration of the possibility that empirical relations found between teacher attributions, emotions, and behaviors may be inflated due to common method variance. More specifically, given that prior studies have primarily employed retrospective self-report techniques to assess teachers’ trait-like causal attributions, as compared to more ecologically valid indicators (i.e., experience sampling measures; Goetz et al., 2015), future research employing more objective, real-time measures of teacher attributions that are less confounded by response (e.g., hindsight) bias are needed to underscore the validity of observed results. Furthermore, an existing overemphasis on cross-sectional methods in prior studies on teachers’ causal attributions necessitates future research efforts in which longitudinal designs are administered (for related research on emotions in educational settings, see Pekrun et al., 2014; Wang et al., 2017) to better elucidate causal relations between teachers’ attributions, emotions, and instructional behaviors.

Finally, initiatives aimed at promoting teacher motivation and well-being are encouraged to incorporate attribution-based interventions to counter teachers’ maladaptive attributional tendencies and biases. One such program is Attributional Retraining (AR); an intervention typically administered to students that encourages causal attributions for failure experiences that are personally controllable in nature, while at the same time discouraging attributions that either absolve responsibility through external attributions or focus instead on internal, uncontrollable factors (e.g., low ability; for reviews, see Haynes et al., 2009). Whether addressing teachers’ attributions for student outcomes (interpersonal) or their own teaching-related setbacks (intrapersonal), attribution-informed interventions such as AR are expected to prove effective in facilitating adaptive changes in teachers’ willingness to assist students (e.g., Tollefson and Chen, 1988; Matteucci et al., 2008b; Woodcock and Jiang, 2013) as well as their own psychological and physical health (e.g., see Hall et al., 2007 for similar AR methods with students; Wang et al., 2015).

## Author Contributions

HW conducted the literature review, screened, synthesized, as well as scholarly wrote the manuscript. NH supervised the project, reviewed and edited the manuscript.

## Conflict of Interest Statement

The authors declare that the research was conducted in the absence of any commercial or financial relationships that could be construed as a potential conflict of interest.
